# Gas Gangrene as Seen at the Casualty Clearing Stations

**Published:** 1917-12

**Authors:** 


***Gas Gangrene As Seen at the Casualty Clearing Stations.***
By Col. CUTHBERT S. WALLACE, C. M. G. Abs. from the *Journal of the Royal Army Medical Corps*, May, 1917.

The author regards gas gangrene as a very striking feature in the surgery of war. Because of its frequency it has ceased to arouse wonder, and is in danger of being accepted as a necessary evil and treated by routine measures. He comments on two articles by D'Este Emery and K. Taylor.

Emery's view he states as follows : " The toxin kills the leucocytes which are the natural protection of the body. To have a sufficient supply of these it is necessary that the circulation be intact. Therefore tissue devitalized by trauma, by constriction of the limb, or by actual damage, will favor the disease. Why, however, does the disease not stop when healthy tissue is reached? The explanation lies in the fact, that the toxin when present in large amount inhibits emigration and kills the leucocytes. If there is no free escape the toxin accumulates to such an extent that it soaks through into the healthy tissue and kills the defensive leucocyte and so the gangrene spreads. "

Taylor's view he states as follows : " The mechanical action of the pressure produced by the gas is usually if not always the most important part of the infection. It brings about the death of the tissues from (*a*) the resulting anemia, (*b*) the actual mechanical fragmentation of the muscle substance, (*c*) the mechanical scattering of the infection. He shows in the early part of his paper that the experimental injection of the exo-toxin produces softening of the muscle and parenchymatous degeneration of the liver cells. His experiments *in vitro* show the same effect upon pieces of muscle. He suggests that the rapid systemic intoxication is produced by the breaking down of the muscle substance, as injection of the exo-toxin does not produce an effect comparable to that seen in the clinical course of the disease."

W. notes that the chief difference between these two conceptions of the disease, lies in the part played by the gas. Taylor considers this the most important factor, while Emery denies this and believes that bacterial toxins are mainly responsible. Emery does not agree with T. that the disease is mainly of the muscles. W. gives his conclusions as follows:

"1. It is rare to meet gas gangrene without a muscle injury.

"2. It is chiefly a disease of the muscles and is rarely dangerous unless muscle is involved.

"3. The lesion in its early stages may be described as a longitudinal one, running up and down the wounded muscles from the seat of the lesion..... Muscles and groups of muscles are involved while others escape.

"4. It is rare to find all the muscles of a segment of a limb involved, save in a segment distal to one in which the main blood supply has been cut off. Thus the whole leg dies and becomes gaseous when the femoral artery has been blocked in the thigh.

"5. The muscles affected are in the first instance the wounded ones. If the pressure caused by the disease is relieved, the gangrene will most probably be confined to these muscles, but if the pressure is not relieved the other muscles may so have their blood supply checked as to fall victims to the infection.

"6. Muscles contained in rigid compartments such as the anterior tibial group are especially prone to die if wounded.

"7. There is but little tendency for the infection to pass from one muscle to another. This is well shown in amputation stumps, where one muscle dies and becomes gaseous, while the rest of the cut muscles remain healthy.

"8. The infection is farther advanced in the muscles than in the intermuscular areolar planes.

"9. The muscles become resonant from the presence of gas long before they become crepitant to the finger, though this phenomenon may be perceptible at an early date by means of the stethoscope....

"10. The presence of gaseous crepitation does not necessarily mean microbic infection....

" 11. Crepitation is usually a comparatively late phenomenon and is due to the escape of gas into the areolar and subcutaneous tissue.

" 12. In an infected limb, a vascular lesion will be followed by the death of the muscle or the muscle group, which death would not have followed in an uninfected limb. It is believed that the pressure produced by the gas so raises the tension in the limb as finally to arrest the circulation.

" 13. In an infected limb there are several conditions of the muscles : (*a*) Normal purple red contractile muscle which may or may not be infected as judged by cultural experiments. (*b*) Dead, non contractile, non-crepitant muscle which has a peculiar red color and is less translucent than normal muscle. (*c*) Dead, non-contractile, crepitant muscle which has the same appearance as the last. (*d*) Brown, black, or diffluent muscle.

" [Muscle dead from the cutting of the blood supply is a purplish brown and its naked eye appearance quite different from (*b*) and (*c*).]

" 14. The microscopic appearances of muscle dead from cutting off its blood supply are different to those of a muscle dead from infection. The striation is present in the former and absent in the latter.

" 15. The bacteria are between the muscle fibres and not in them.

" 16. — Microscopical examination suggests that the gas may find its way between the muscle fibers in front of the bacterial invasion.

" 17. In dead infected muscles the fibers are separated from one another. This separation is more marked in muscles that are crepitant than in those that have not yet reached that stage. "

W. further calls attention to the fact that muscles with an intact blood supply are also liable to be killed, although the method of their death is not clear. The way in which the infection spreads also is not known. The author believes that pressure is a great factor, but he is uncertain as to whether it acts wholly by cutting off the main blood supply or by allowing the gas to penetrate the muscles and produce an anemia of the individual fibers, or by favoring the penetration of the still living muscle by toxins derived directly or indirectly from the bacilli.

The extension of the infection W. believes may be brought about in two ways:

1. *The toxins produced by the Bacteria.* — Most striking in microscopic sections of the muscles dead of gas infection is the loss of striation and the breaking up of the muscle fibre substance. These appearances are quite different from those seen in uninfected dead muscles. W. believes there must be some reason for this difference other than the action of gas or the presence of the bacillus, because it appears often to the same extent in microscopic sections in which few or many bacilli are present, and in which there is much or little distension due to the action of gas. He adds: "The change in the muscle fiber may, therefore, be due either to some toxin produced directly or indirectly by the bacteria." W. is inclined to agree withe Taylor's theory that toxin s produced by disintegration of the muscle substance by bacterial action may supplement the action of the exo-toxins of the organism, and W. adds that the toxic muscle substance produced in the traumatized portion of the muscle may be carried into the more distal parts of the muscle and cause its death.

2. *The part played by the Gas.* — Regarding the part played in the limb, W. believes that nothing but the rapid evolution of gas could possibly account for the tense and tympanitic state of the limb, and the rapidity with which this condition is reached. Although gas may be present in the areolar planes, it is not usually the case to any degree. Crepitation is by no means always evident when gas is present, for it has been found that muscle may be dead and full of gas, and yet not crepitant to the finger. Gas in the areolar tissue follows the path of the least resistance, and often spreads into the subcutaneous tissue a considerable way from the wound. Only wounded muscle yields to the infection in the first instance, but muscle so infected may injure neighboring muscles by exerting on them a high pressure and checking their circulation. Gross infection of the areolar planes could not produce such an effect, because the sugar of the muscle is needed for the gas production. W. agrees with Taylor that the action of the gas may lead to the disintegration of the muscle fiber substance, and he thinks that it also may drive the toxins into the circulation, and thus be a factor in the systemic poisoning. W. also agrees with Taylor as against Emery that the formation of gas is essential to the spread of the disease, because clinical experience in a Casualty Clearing Station is opposed to Emery's statement that "gangrene may occur and the patient die without the formation of any gas in the tissues." W. has noticed no suppuration in fatal cases in the clearing stations.

W. believes that either extension along the blood vessels or systemic infection may be responsible for metastases.

(The remainder of this article is abstracted under "Treatment", p. 128).

